# Cystic Lymphangioma of the Rectus Abdominis Muscle: An Extremely Rare Clinical Entity

**Published:** 2014-07-19

**Authors:** Saloua Ammar, Mohamed Jallouli, Mahdi Ben Dhaou, Riadh Mhiri

**Affiliations:** Department of Pediatric Surgery, Hedi Chaker Hospital, School of Medicine, Sfax University, Sfax, Tunisia

**Keywords:** Lymphangioma, Cysts, Rectus Abdominis Muscle

Cystic lymphangioma is a rare malformative benign tumor of the lymphatic vessels. It occurs most commonly in the neck. Rarely they are known to involve the axilla, groin, mediastinum, retroperitoneum, pelvis, mesentery, omentum and spleen^[^^1^^]^. A 10 year old girl, without any particular pathological history, presented with complaints of swelling in the left side of abdomen increasing very slowly in size since the age of 3 years. Physical examination objectified left parietal bulk, compressible, by 5 cm long axis without signs of acute inflammation. Abdominal US showed a left paramedian parietal mass sub umbilical developed at the expense of the rectus abdominis muscle. This mass was well limited, measuring 57×41 mm, hypoechoic, micro cystic with many thin septa. She arrived deeply in contact with bowel loops and iliac vessels.

**Fig. 1:**
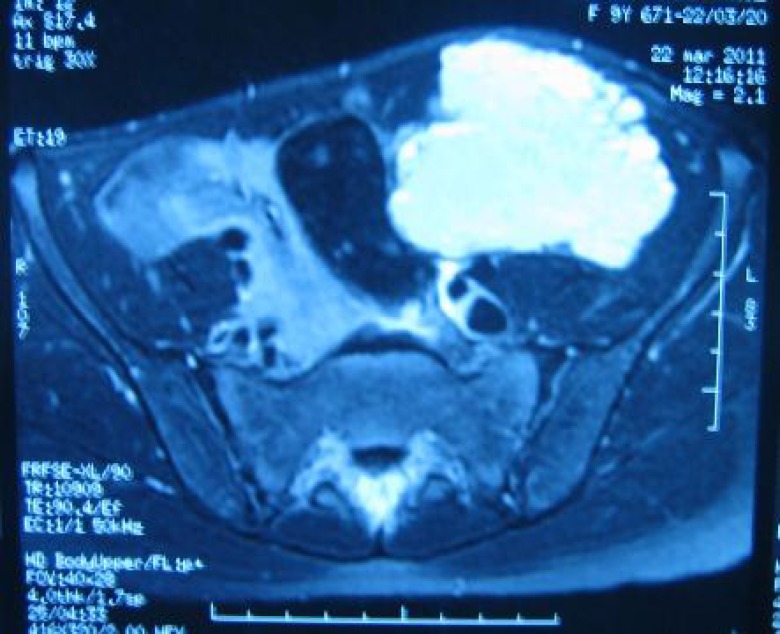
Abdominal MRI: hypersignal mass developed at the expense of the rectus abdominis with a contact with the psoas muscle, the sigmoid colon and the external iliac pedicle

 Further exploration by MRI showed a polylobed mass, developed at the expense of the rectus abdominis muscle. It extended to the large muscles of the ipsilateral side, with a hypo-signal T1 and hyper-signal T2. This formation was responsible for ascension of small bowel and came in contact with the external iliac pedicle, the left psoas muscle and the sigmoid without invasion. The MRI appearance was compatible with a cystic lymphangioma. Per operatively, the cystic mass was attached at the rectus abdominis muscle (Fig. 1). Entire dissection and excision of the mass sacrificing a musculofacia piece and a tab of the parietal peritoneum were performed. There was no adhesion with the iliac vessels. 

 Histological examination revealed an intramuscular cystic lymphangioma extending to the peritoneum. The postoperative course was uneventful. Subsequent control after 10 months showed a child in remission.

 Cystic lymphangioma is a rare congenital malformation. The failure of connection or separation between the lymphatic and venous system and the proliferation of abnormal lymphatic tissue are two theories most implicated in its development^[^^2^^]^. Most of the cystic lymphangiomas are discovered during childhood; however, their nascent presentation in adults is also reported^[^^3^^]^.

 It can manifest anywhere in the body. The common locations are cervico-facial regions. Muscle *location, extremely scarse*, was described both in adults^[^^3^^]^ and children^[^^4^^]^ but not in the rectus abdominis muscle. The clinical presentation of cystic lymphangioma is polymorphic, unspecific and variable depending on the location and extent.

 In front of a cystic mass localized in the muscle several diagnostics can be evoked, such as hematoma or cystic muscle remodeling especially following trauma or tumor lesions or muscle abscess^[^^3^^]^. Imaging in our case was highly suggestive of the diagnosis. US^[^^5^^]^ and MRI^[^^3^^,^^5^^,6]^ are *excellent ways for diagnosis* of cystic lymphangiomas in several locations. US usually shows multicystic lesion with internal septations and no blood flow is detected on color doppler ultrasonographs. Other modalities like CT scan and MRI can be employed to delineate the lesion, in a better way. A CT scan demonstrates multicystic, homogeneous, non-invasive density with low attenuation. These modalities are usually helpful in ascertaining the extent of the lesion and their association with nerves and vessels and are particularly useful, when surgical management of the lesion is contemplated^[^^2^^]^.

 MRI of lymphangioma usually shows a multiloculated heterogeneous mass with low signal intensity on T1-weighted images, and high signal intensity on T2-weighted images because of its content. The authors' experience suggests that most lymphangiomas have a characteristic appearance on MRI. The information obtained with MRI can help in providing a preoperative diagnosis, in planning surgical resection, and in defining recurrence^[^^5^^]^.

 A total surgical excision, if feasible without a major sacrifice of adjacent organs, can be the best therapeutic option^[^^3^^,6]^. Sclerotherapy presents an interesting therapeutic method in invasive forms^[^^1^^]^. In our patient complete resection was performed. The anatomopathologic study confirmed the diagnosis. Postoperative course was simple.

 The presentation of this case draws attention to the possibility of occurrence of such an etiologic lesion in rectus abdominis muscle. We stress the necessity of imaging as a diagnostic tool for this location. 
